# A simple scoring system to estimate adenoma detection rate from polyp detection: a two-center observational study

**DOI:** 10.1186/s12876-026-04984-2

**Published:** 2026-06-02

**Authors:** Zhiyu Dong, Yanglei Li, Zili Xiao, Feng Li, Shuchang Xu, Danian Ji

**Affiliations:** 1 Department of gastrointestinal endoscopy, Huadong Hospital, Fudan University, Shanghai, China; 2 Department of Gastroenterology, Tongji Hospital, Tongji University School of Medicine, Shanghai, China

**Keywords:** Adenoma detection rate, Polyp detection rate, Scoring system, Colonoscopy

## Abstract

**Purpose:**

The calculation of adenoma detection rate (ADR) requires pathological information, which makes it time-consuming. We aimed to develop a scoring system to estimate ADR from polyp detection accurately.

**Methods:**

The two-center retrospective study included colonoscopy and pathological data, which were divided into training, internal, and external validation cohorts. The factors associated with pathologically confirmed adenoma detection in patients with polyps were identified using the logistic regression model. The scores were assigned based on β-coefficient. The performance of the scoring system was evaluated by the Bland-Altman plot.

**Results:**

Patient age, maximum size of polyps, and number of proximal or distal polyps of any size, except distal diminutive polyps, were significantly associated with pathologically confirmed adenoma detection in patients with polyps. Based on regression coefficients, scores were assigned as follows: age ≥ 70 years, positive; any proximal polyps, positive; distal polyps ≥ 10 mm, positive; age 60 ~ 69 years, 1 point; per distal small polyps, 1 point. Pathologically confirmed adenoma detection was predicted when a patient met any positive criterion or, in the absence of these positive items, had a total score > 1 point. The scoring system showed a high proportion of acceptable estimations when the difference between estimated and actual ADR was less than 2%. In clinically relevant conditions with a small number of samples, the proportion of acceptable estimations rose gradually and achieved its highest level when the number of samples increased to 300.

**Conclusion:**

Our scoring system performed well in estimating ADR from polyp detection. 300 colonoscopies per endoscopist was the best number of samples when the scoring system was used.

## Introduction

Colorectal cancer (CRC) is the third most common cancer worldwide and the second leading cause of cancer-related death, with approximately 1.9 million new cases and more than 900,000 deaths in 2022 [1]. Many studies have shown that colonoscopy reduces CRC incidence and mortality, therefore it is currently considered as gold standard for CRC screening [2, 3]. ADR has been found to be associated with lower interval CRC and the European Society Gastrointestinal Endoscopy (ESGE) and British Society of Gastroenterology have identified ADR as a validated performance measure and “ADR ≥ 25% in patients aged 50 years or older” as well as “ADR ≥ 15% in general all patient population” as minimum standard for endoscopists [4–6]. However, the calculation of ADR requires both endoscopic findings and histological information, but the automatic link between information is not easily implemented in many medical units, which makes the calculation of ADR a time-consuming and labor-intensive undertaking.

Polyp detection rate (PDR) has been found to be correlated with ADR and non-inferior to ADR in predicting the risk of interval CRC [7, 8]. When histological information is not easily combined with endoscopic data, ESGE suggests that PDR could be a surrogate for ADR [5]. Furthermore, the UK and Ireland working group has proposed that PDR might be considered as a useful surrogate for ADR once the validity is confirmed for the units [6]. In addition, previous studies demonstrated that overall adenoma to polyp detection rate quotient (APDRQ) is a useful conversion factor to accurately estimate the ADR from the PDR [9, 10], and the conversion factor 0.64 was recommended in latest ESGE guideline. However, the patient-related and polyp-related features associated with pathologically confirmed adenoma detection in patients with polyps were not taken into account in conversion method in these studies and whether scoring systems developed by these features are superior to APDRQ in clinical practice is still unknown.

The aim of this study was to develop scoring system based on patient-related and polyp-related features and compare the performance in clinical practice between our scoring system and conversion factor (0.64) recommended by ESGE guideline. In this study, the screening and surveillance colonoscopy and corresponding pathological data from multi-center were retrospectively included and the data were further divided into training, internal validation and external validation cohorts. The significant patient- and polyp-related features associated with pathologically confirmed adenoma detection in patients with polyps were identified using logistic regression model in training cohort and the scoring system was developed based on the regression coefficients. Then, the performance of scoring system in estimating ADR from PDR in each endoscopist was compared with recommended conversion factor (0.64) using Bland-Altman plot.

## Materials and methods

### Study design and population

All colonoscopy data were retrospectively collected from endoscopic procedure database at Huadong hospital (From August, 2012 to August, 2023) and Tongji Hospital (From January, 2012 to June, 2019) in Shanghai, China. Patient age and gender, bowel preparation quality, sedation use and endoscopic manifestation were all documented. Polyp characteristics such as location and size were extracted from endoscopic reports and the histological information of polyps was obtained from another electronic database in these two hospitals.

**Fig. 1:**
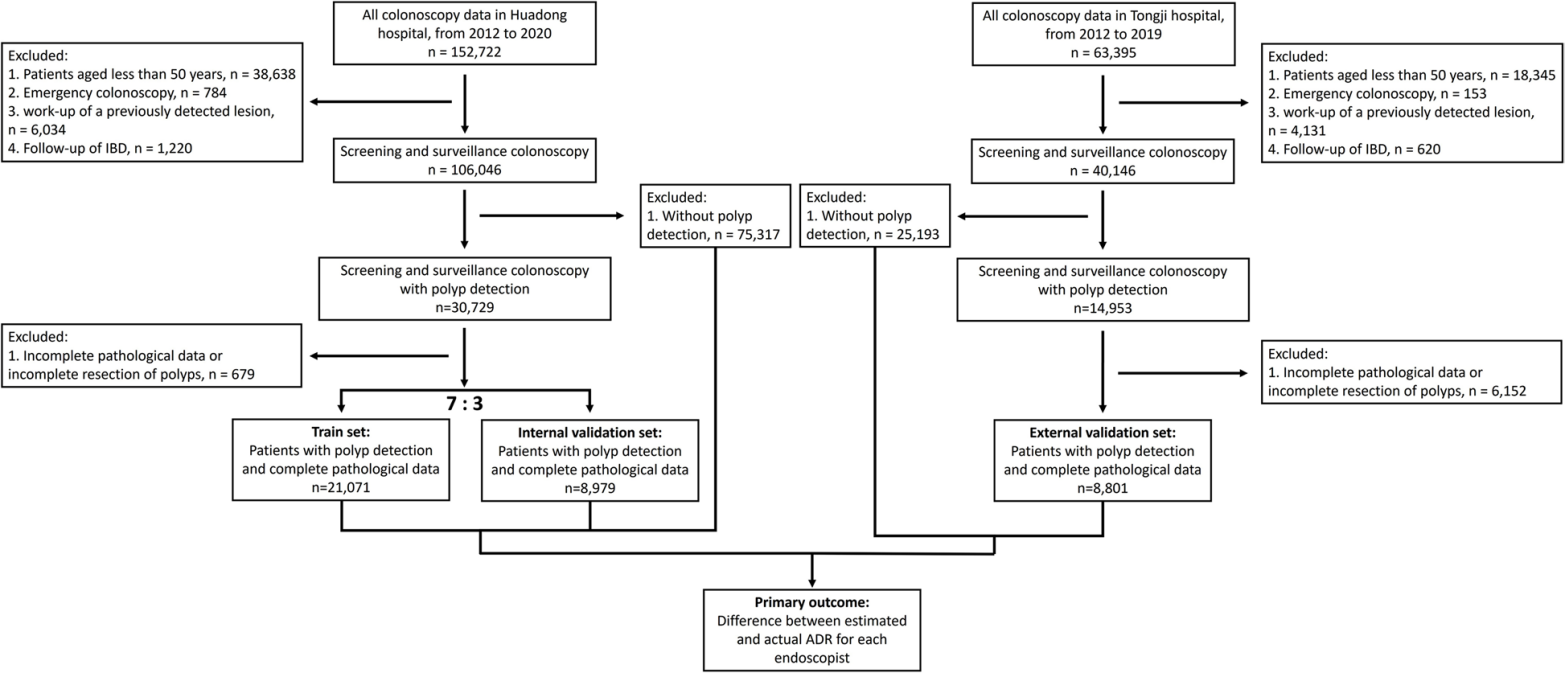
Flow chart of the study population

Figure 1 shows the flowchart of study participant selection. According to the population used to calculate ADR and PDR in ESGE guideline, the exclusion criteria in this study were as follows: (1) Patients aged less than 50 years (2) Emergency colonoscopy (3) colonoscopy for work-up of a previously detected lesion (4) colonoscopy for follow-up of inflammatory bowel disease or other chronic disease (5) colonoscopy with incomplete resection of polyps or incomplete pathological data.

Totally, 30,050 colonoscopies with complete resection of polyps and complete pathological data in Huadong hospital were included and randomly allocated into training and internal validation cohort at a ratio of 7 to 3, in order to develop the scoring systems and validate their predictive value, respectively. 8801 colonoscopies with complete resection of polyps and complete pathological data in Tongji hospital were included as external validation cohort to validate the predictive value and generalization of these scoring systems.

In addition, 75,317 and 25,193 colonoscopies without polyp detection in Huadong hospital and Tongji hospital were also included as negative cases for the analysis of the performance of developed scoring systems in estimating ADR from PDR in each endoscopist.

The Tongji Hospital Ethics Committee approved this study(K-W-2020-008). This study was a retrospective and observational study, and patient consent is not signed by each patient.

### Developing the scoring system and setting the optimal cut-off

#### Identification of significant factors

In this study, patient-related features including age and gender and polyp-related features including number, location and size were included. Due to the different proportion of pathologically confirmed adenoma in polyps located in proximal and distal colon and in polyps with different size, we further integrated the polyp-related features into the following factors: the number of proximal polyps, the number of distal polyps, the maximum size of proximal polyps, the maximum size of distal polyps, the number of diminutive, small or intermediate/large polyps in proximal colon and the number of diminutive, small or intermediate/large polyps in distal colon. In this study, diminutive polyps were defined as polyps ≤ 5 mm, small polyps as 6–9 mm, and intermediate/large polyps as ≥ 10 mm.

The factors independently associated with pathologically confirmed adenoma detection in patients with polyp detection were identified using logistic regression model. In order to avoid multicollinearity, the number of proximal or distal polyps and the maximum size of proximal or distal polyps were included into logistic regression model as Model 1, while the number of diminutive, small or ≥ 10 mm polyps in proximal or distal colon were entered into logistic regression model as Model 2.

#### Assigning score for significant factors

The scores were assigned for each factor based on β-coefficient obtained from logistic regression model. In each logistic regression model, scores were assigned to each significant factor by dividing the β-coefficient by the median of β-coefficients of all significant factors. Then, two scoring systems for each model was developed using the following manners: (1) score 1, when the decimal part of score falls within the range of 0 to 24, 25 to 74 and 75 to 99, it is approximated as 0, 50 and 100, respectively. (2) score 2, when the decimal part of score falls within the range of 0 to 49 and 50 to 99, it is approximated as 0 and 100, respectively.

#### Setting the optimal cut-off for each scoring system

The cut-off for each scoring system was set based on the performance of scoring systems in predicting pathologically confirmed adenoma detection in patients with polyp detection in training cohort. The best cut-off for each scoring system was chosen when the lowest deviation between the estimated and actual ADR in patients with polyp detection was got in training cohort.

### Study outcomes

#### Primary outcome measure

The primary outcome of this study was the agreement between estimated and actual ADR in each endoscopist, as well as the proportion of accurate predictions, defined as an absolute difference of less than 2% points between estimated and actual ADR. This threshold was prespecified by the investigators as a pragmatic criterion for endoscopist-level assessment, based on the consideration that this corresponds to only a small deviation from the guideline-recommended minimum ADR benchmark of 25%.

#### Secondary outcome measure

The secondary outcomes of this study included the predictive value of scoring system in predicting pathologically confirmed adenoma detection in patients with polyp detection, inaccuracy rate and deviation rate between estimated and actual ADR in patients with polyp detection.

The inaccuracy rate was defined as dividing the sum of false-positive and false-negative subjects by all subjects. The deviation rate was defined as dividing absolute value of the difference between false-positive and false-negative subjects by all subjects.

### Assessment of performance of the scoring system

#### Receiver operating characteristics (ROC) curve analysis

To evaluate the predictive value of the scoring system in estimating pathologically confirmed adenoma detection in patients with polyp detection, receiver operating characteristics (ROC) curve analysis was performed to calculate the area under the ROC curve (AUROC). When the AUROC falls within the range of 0.7 to 0.9 and 0.9 to 1, it represents moderate and high predictive value, respectively.

#### Bland-Altman method

Bland-Altman plot is a well-known and widely used method for describing agreement between two quantitative measurements [11]. In brief, the difference between the two paired quantitative measurements is plotted against the average of these two measurements. The 95% limit of agreement (LOA) of Bland-Altman plot is identified as the confidence limits of the mean difference between two measurements, which represents that 95% of the difference between two measurements should lie within LOA. A maximum acceptable difference based on biologically or clinically relevant criteria is defined prior and relationship between maximum acceptable difference and LOA is one of the most important indicators for agreement between two measurements in Bland-Altman plot.

#### Random sampling for investigating the generalization and practical significance

The small-sample analysis was performed to evaluate the generalizability and practical applicability of the scoring system under clinically relevant conditions with limited case volumes. For each predefined sample size of 100, 200, 300, and 400 procedures per endoscopist, random sampling without replacement was conducted among endoscopists with sufficient case numbers. For each sample size, the sampling procedure was repeated 10 times for each eligible endoscopist.

In each simulated subset, the actual ADR and estimated ADR were calculated at the endoscopist level. The difference between estimated and actual ADR was calculated for each endoscopist, and the mean difference was derived for each simulation. Across the 10 repeated simulations for each sample size, the average mean difference and its 95% confidence interval were reported. The proportion of acceptable estimations, defined as the proportion of endoscopists whose estimated ADR fell within ± 2% and ± 3% of the actual ADR, was calculated for each simulation. The mean proportion across the 10 simulations and its 95% confidence interval were presented.

### Sample size estimation

The sample size estimation was based on the principle of 10 outcome events per variable. In this study, two independent logistic regression models were used to develop scoring systems. In Model 1 and Model 2, a maximum of 4 and 8 variables will be included in the final regression model. Thus, this study required at least 80 pathologically confirmed adenoma detection in patients with polyp detection in training cohorts.

### Statistical analysis

R language version 4.3.0 (R Foundation for Statistical Computing, Vienna, Austria) was used for data analysis. Continuous variables were expressed as mean ± standard deviation for normally distributed variables or median [interquartile range] if the data were not normally distributed. Categorical variables were presented as number (n) and percentage (%). The student t-test and Chi-square test were used to compare the clinical characteristics between subjects in training and internal validation cohorts, as well as subjects in different centers. The Chi-square test was used to compare the proportion of acceptable estimations between developed scoring system and recommended conversion factor (0.64).

All reported *P* values were two-sided, with *P* < 0.05 defined as statistical significance.

## Results

A total of 30,050 colonoscopies with complete resection of polyps and complete pathological data in Huadong hospital were included and randomly allocated into training cohort (*n* = 21,071) and internal validation cohort (*n* = 8,979). 8801 colonoscopies with complete resection of polyps and complete pathological data in Tongji hospital were included as external validation cohort.

The clinical characteristics of all subjects in training and validation cohorts were shown in Table 1. There were no significant differences in age, gender, sedation, bowel preparation, as well as number, maximum size, location-distribution, size-distribution of polyps between training and internal validation cohort. However, there were significant differences in all patient- and polyp-related characteristics between subjects in Huadong hospital and Tongji hospital, which demonstrates the heterogeneity in different validation cohorts and considers the external validation cohort suitable for exploring the generalization of scoring system.

**Table 1:** Clinical characteristic of included patients received colonoscopy in Huadong hospital and Tongji hospital

	Huadong hospital			Tongji hospital	
	Train set (*n* = 21071)	Internal validation set (*n* = 8979)	*p*-value^a^	External validation set (*n* = 8801)	*p*-value^b^
Age (mean (SD))	64.42 (8.45)	64.46 (8.46)	0.709	64.03 (8.49)	< 0.001
Male (n(%))	12,799 (60.7)	5519 (61.5)	0.245	4543 (51.6)	< 0.001
Sedation (n(%))	14,001 (66.4)	5997 (66.8)	0.574	7343 (83.4)	< 0.001
Poor bowel preparation (n(%))	906 (4.30)	383 (4.30)	0.918	280 (3.20)	< 0.001
Number of polyps (median [IQR])	1.00 [1.00, 2.00]	1.00 [1.00, 2.00]	0.065	1.00 [1.00, 2.00]	< 0.001
Maxsize of polyps (median [IQR])	0.60 [0.50, 1.00]	0.60 [0.50, 1.00]	0.052	0.60 [0.40, 1.20]	< 0.001
Location of polyps					
Cecum or ascending colon (n(%))	6628 (31.5)	2771 (30.9)	0.315	1478 (16.8)	< 0.001
Transverse colon (n(%))	7444 (35.3)	3165 (35.2)	0.906	2666 (30.3)	< 0.001
Descending colon (n(%))	2870 (13.6)	1223 (13.6)	1.000	293 (3.30)	< 0.001
Sigmoid colon (n(%))	7292 (34.6)	3014 (33.6)	0.085	2538 (28.8)	< 0.001
Rectum (n(%))	4815 (22.9)	2020 (22.5)	0.512	1988 (22.6)	0.768
Size of polyps					
Diminutive polyps(n(%))	11,419 (54.2)	4879 (54.3)	0.827	4056 (46.1)	< 0.001
Small polyp (n(%))	8695 (41.3)	3691 (41.1)	0.809	2975 (33.8)	< 0.001
Intermediate or large polyps (n(%))	5699 (27.0)	2346 (26.1)	0.103	2799 (31.8)	< 0.001
Adenoma detection (n(%))	16,869 (80.1)	7183 (80.0)	0.917	6516 (74.0)	< 0.001
Advanced adenoma detection (n(%))	3459 (16.4)	1449 (16.1)	0.562	1893 (21.5)	< 0.001
Colorectal cancer detection (n(%))	1516 (7.20)	668 (7.40)	0.469	1361 (15.5)	< 0.001

^a^comparison between train set and internal validation set

^b^comparison between all data from Huadong hospital and external validation set

### The proportion of pathologically confirmed adenoma in different-sized polyps and polyps located in different segments of colon

In data from training and internal validation cohorts, the proportion of pathologically confirmed adenoma in diminutive, small, intermediate and large polyps located in proximal colon were 0.738, 0.786, 0.819 and 0.947, respectively. This proportion was similar in small, intermediate and large polyps located in distal colon (0.740, 0.868 and 0.982, respectively). However, the proportion in distal diminutive polyps decreased to 0.514, which differed largely from the proportion in proximal diminutive polyps (Table 2).

**Table 2:** The proportion of pathologically confirmed adenoma in polyps included

	Size of polyps			
	Diminutive	Small	Intermediate	Large
Huadong Hospital				
Location of polyps				
Cecum or ascending colon	0.722	0.754	0.793	0.955
Transverse Colon	0.752	0.808	0.844	0.936
Descending Colon	0.705	0.820	0.850	0.977
Sigmoid Colon	0.565	0.764	0.879	0.978
Rectum	0.377	0.625	0.856	0.985
Proximal Colon	0.738	0.786	0.819	0.947
Distal Colon	0.514	0.740	0.868	0.982
Tongji Hospital				
Location of polyps				
Ileocecum or ascending colon	0.661	0.800	0.815	0.863
Transverse Colon	0.685	0.779	0.820	0.860
Descending Colon	0.603	0.775	0.837	0.922
Sigmoid Colon	0.435	0.722	0.859	0.925
Rectum	0.268	0.612	0.814	0.913
Proximal Colon	0.677	0.786	0.818	0.862
Distal Colon	0.369	0.689	0.840	0.919

In data from external validation cohort, the proportion of pathologically confirmed adenoma in diminutive, small, intermediate and large polyps located in proximal colon were 0.677, 0.786, 0.818 and 0.862, respectively. The proportion in diminutive, small, intermediate and large polyps located in distal colon were 0.369, 0.689, 0.840 and 0.919, respectively (Table 2).

These results indicate that the proportion of pathologically confirmed adenoma varies largely in polyps located in proximal and distal colon, especially in proximal and distal diminutive polyps.

### Development of scoring systems and setting of optimal cut-off for each scoring system

Table 3 showed two independent logistic regression models for predicting pathologically confirmed adenoma detection in patients with polyp detection. Model 1, which enrolled age, gender and polyp-related features including the number of polyps and maximum size of polyps, demonstrated that age, the number of proximal polyps, the number of distal polyps, maximum size of proximal polyps and maximum size of distal polyps were all significant predictors for pathologically confirmed adenoma detection in patients with polyp detection.

**Table 3:** Logistic regression of pathologically confirmed adenoma and assigned score for the risk factor

	OR （95% CI）	*p*-value	Coefficient	Score 1	Score 2
Model 1					
Age					
50~59 years	Reference	-			
60~69 years	1.14 (1.05-1.24)	0.002	0.13	0	0
≥70 years	1.62 (1.46-1.79)	<0.001	0.48	0.5	1
Male	1.06 (0.98-1.14)	0.152	0.05	0	0
Number of proximal polyps	1.42 (1.32-1.52)	<0.001	0.35	0.5	0
Number of distal polyps	0.91 (0.88-0.94)	0.001	-0.10	0	0
Proximal polyps					
No polyps	Reference	-			
Maxsize of polyps (≤5mm）	2.36 (2.01-2.77)	<0.001	0.86	1	1
Maxsize of polyps (6-9mm)	2.60 (2.19-3.09)	<0.001	0.95	1.5	1
Maxsize of polyps (≥10mm)	4.04 (3.27-5.00)	<0.001	1.40	2	2
Distal polyps					
No polyps	Reference	-			
Maxsize of polyps (≤5mm)	1.84 (1.60-2.12)	<0.001	0.61	1	1
Maxsize of polyps (6-9mm)	4.98 (4.25-5.84)	<0.001	1.60	2	2
Maxsize of polyps (≥10mm)	21.2 (17.1-26.3)	<0.001	3.06	4	4
Model 2					
Age					
50~59 years	Reference	-			
60~69 years	1.15 (1.06-1.24)	0.001	0.14	0.5	0
≥70 years	1.65 (1.49-1.82)	<0.001	0.50	1	1
Male	1.06 (0.99-1.14)	0.099	0.06	0	0
Proximal colon					
Number of diminutive polyps	1.65 (1.55-1.75)	<0.001	0.50	1	1
Number of small polyps	1.74 (1.63-1.86)	<0.001	0.55	1	1
Number of polyps ≥10mm	1.96 (1.76-2.17)	<0.001	0.67	1.5	1
Distal colon					
Number of diminutive polyps	0.89 (0.86-0.93)	<0.001	-0.11	0	0
Number of small polyps	1.44 (1.36-1.54)	<0.001	0.37	0.5	1
Number of polyps ≥10mm	4.61 (4.02-5.28)	<0.001	1.53	3	3

Model 2, which enrolled age, gender and polyp-related features including the number of diminutive, small and ≥ 10 mm polyps, demonstrated that age and the number of proximal and distal polyps with any size, except the distal diminutive polyps, were all significant predictors for pathologically confirmed adenoma detection in patients with polyp detection.

Based on the regression coefficients and the method of approximation in Score 1 and Score 2, we assigned scores for each significant predictor in Model 1 and Model 2. The detailed score for each predictor was shown in Table 3.

**Fig. 2:**
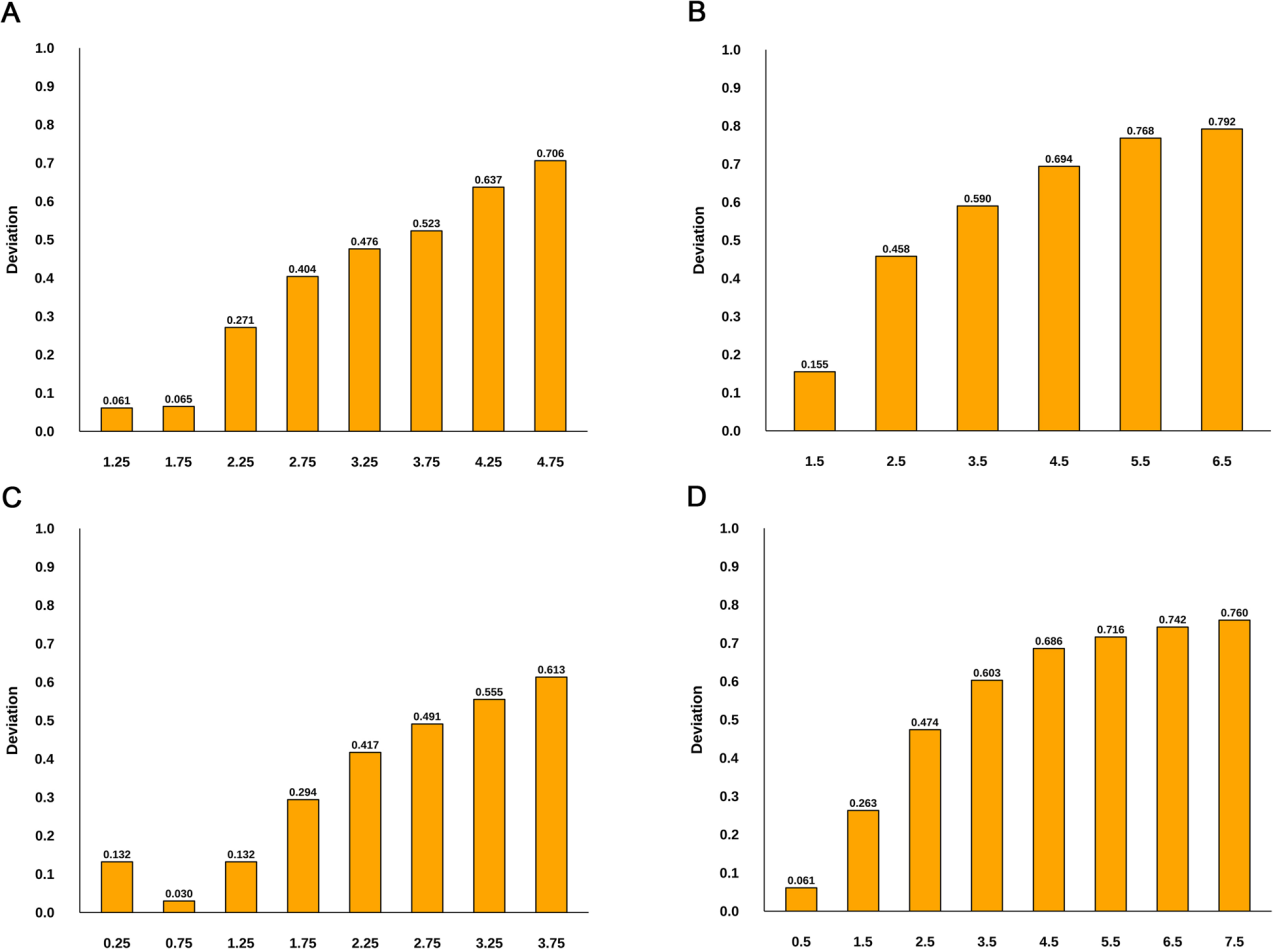
The deviation between estimated and actual ADR in patients with polyp detection in training cohort when the different cut-off was used. **A** estimated by Model 1 score 1 (**B**) estimated by Model 1 score 2 (**C**) estimated by Model 2 score 1 (**D**) estimated by Model 2 score 2. ADR, adenoma detection rate

Figure 2 showed the deviation between estimated and actual ADR in patients with polyp detection when different cut-off was used. In order to get a lowest deviation between estimated and actual ADR in patients with polyp detection in training cohort, the cut-off of Score 1 and Score 2 in Model 1 and Model 2 was set as 1.25, 1.5, 0.75 and 0.5, respectively.

### Performance of scoring systems in predicting pathologically confirmed adenoma detection in patients with polyp detection

**Fig. 3:**
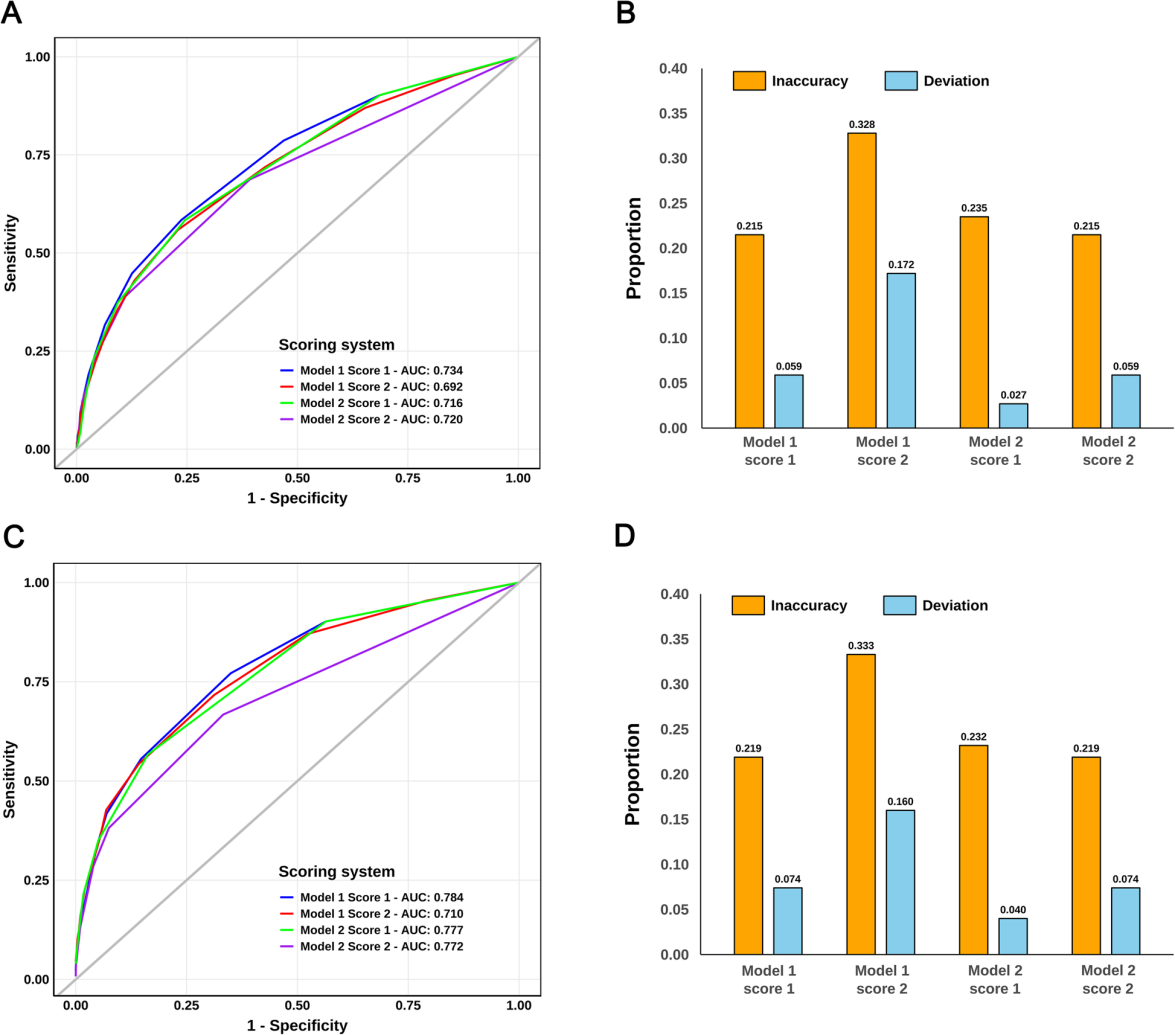
The performance of 4 optional scoring systems in estimating adenoma detection in patients with polyp detection in validation cohorts (**A**) ROC curves for predicting adenoma detection in patients with polyp detection in internal validation cohort (**B**) The inaccuracy rate and deviation between estimated and actual ADR in patients with polyp detection in internal validation cohort (**C**) ROC curves for predicting adenoma detection in patients with polyp detection in external validation cohort (**D**) The inaccuracy rate and deviation between estimated and actual ADR in patients with polyp detection in external validation cohort. ADR, adenoma detection rate. ROC, receiver operating characteristic

The Model 1 score 1, Model 2 score 1 and Model 2 score 2 showed moderate predictive value in predicting pathologically confirmed adenoma detection in patients with polyp detection in both internal validation cohort (Model 1 score 1, AUC = 0.734; Model 2 score 1, AUC = 0.716; Model 2 score 2, AUC = 0.720) and external validation cohort (Model 1 score 1, AUC = 0.784; Model 2 score 1, AUC = 0.777; Model 2 score 2, AUC = 0.772) (Fig. 3A and C).

In addition, Model 1 score 1, Model 2 score 1 and Model 2 score 2 showed similar inaccuracy in predicting pathologically confirmed adenoma detection in patients with polyp detection in both internal validation cohort (Inaccuracy: Model 1 score 1, 0.215; Model 2 score 1, 0.235; Model 2 score 2, 0.215) and external validation cohort (Inaccuracy: Model 1 score 1, 0.219; Model 2 score 1, 0.232; Model 2 score 2, 0.219). As for deviation between estimated and actual ADR in patients with polyp detection, Model 2 score 1 showed the lowest deviation in both internal validation cohort (0.027) and external validation cohort (0.04) (Fig. 3B and D).

Thus, Model 2 score 1 was selected as the final scoring system for further analysis. According to the score assigned to each factor and the optimal cut-off, this model was further simplified into a 5-item scoring system: (i) age ≥ 70 years, positive; (ii) any proximal polyp, positive; (iii) any distal polyp ≥ 10 mm, positive; (iv) age 60–69 years, 1 point; and (v) each distal small polyp, 1 point (Table 4). In the simplified scoring system, “positive” was defined as the presence of at least one of the first three items. Therefore, pathologically confirmed adenoma detection was predicted when a patient had any positive item or, if none of these positive items was present, had a total score > 1 point.

**Table 4:** Simplified scoring systems

Items	Score
Age ≥ 70 years	Positive
Proximal polyps (any size)	Positive
Distal polyps (≥ 10 mm)	Positive
Age (60 ~ 69 years)	1
Per distal small polyps	1

### Comparing the level of agreement between actual and estimated ADR in each endoscopist

**Fig. 4:**
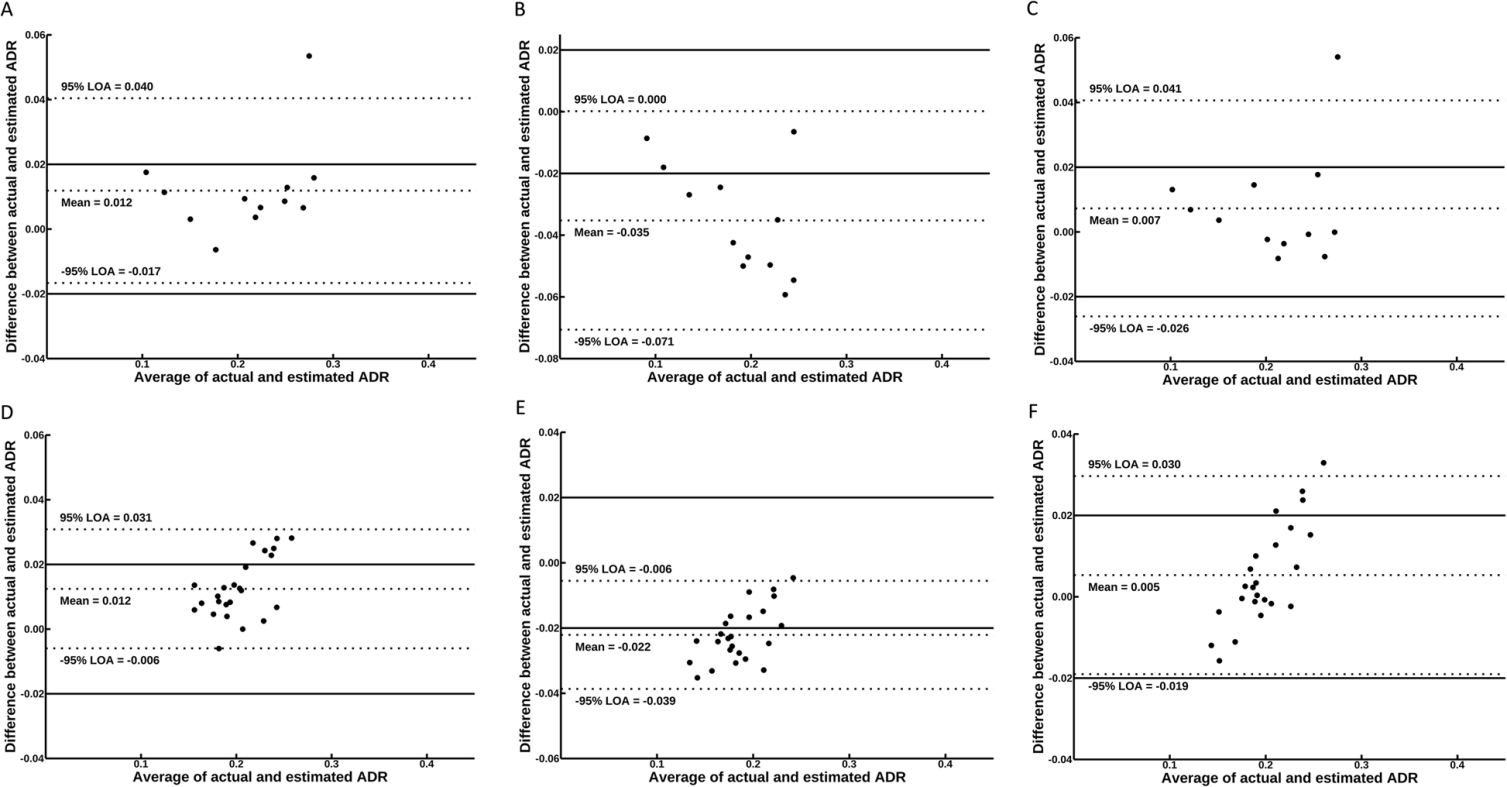
The Bland-Altman plot for difference between estimated and actual ADR for each endoscopist against the average of two measurements. **A-C** the performance of our developed scoring system, recommended conversion factor (0.64), and institution-specific conversion factor in endoscopists from Huadong hospital. **D-F** the performance of our developed scoring system, recommended conversion factor (0.64), and institution-specific conversion factor in endoscopists from Tongji hospital. The horizontal dotted lines were mean and 95% limit of agreement. The horizontal full lines were the maximum acceptable difference (± 2%). ADR, adenoma detection rate; LOA, limit of agreement

In order to evaluate the level of agreement between actual and estimated ADR, we used Bland-Altman analysis in this study. In data from training and internal validation cohort, the mean difference (-95% LOA to 95% LOA) between estimated ADR calculated by our developed scoring system, conversion factor (0.64), and institution-specific conversion factor, and actual ADR were 1.2% (-1.7% to 4.0%), -3.5% (-7.1% to 0.0%), and 0.7% (-2.6% to 4.1%), respectively (Fig. 4A-C). In data from external validation cohort, the mean difference (-95% LOA to 95% LOA) between estimated ADR calculated by our developed scoring system and conversion factor (0.64), and institution-specific conversion factor, and actual ADR were 1.2% (-0.6% to 3.1%), -2.2% (-3.9% to -0.6%), and 0.5% (-1.9% to 3.0%), respectively (Fig. 4D-F). In addition, the proportion of acceptable estimations when the difference between estimated and actual ADR was less than 2% was comparable between our developed scoring system and institution-specific conversion factors, and both methods significantly outperformed the conversion factor (0.64) (Our scoring system vs. conversion factor (0.64) vs. institution-specific conversion factor, Huadong cohort: 91.7% vs. 25.0% vs. 91.7%, *p* < 0.001; Tongji cohort: 75.0% vs. 37.5% vs. 83.3%, *p* = 0.003).

### Exploring the generalization and practical significance of scoring system

In order to explore the generalization and practical significance of our developed scoring system, the performance of which in condition with small number of samples were further investigated (Table 5).

**Table 5:** Results of level of agreement in Bland-Altman analysis in condition with small number of samples

	Mean difference (95% CI)	Proportion of deviation ≤ 2% (95% CI)	Proportion of deviation ≤ 3% (95% CI)
Number of samples: 100			
Scoring system	1.28% (1.00% − 1.56%)	56.7% (51.3% − 62.1%)***	78.9% (73.0% − 84.8%)***
Conversion factor (0.64)	-2.47% (-2.75% - -2.20%)	40.0% (35.3% − 44.7%)	60.8% (53.2% − 68.5%)
Institution-specific conversion factors	0.74% (0.46% − 1.03%)	58.3% (52.4% − 64.3%)***	78.9% (74.8% − 83.0%)***
Number of samples: 200			
Scoring system	1.40% (1.17% − 1.63%)	66.4% (60.9% − 71.9%)***	82.8% (80.2% − 85.4%)***
Conversion factor (0.64)	-2.58% (-2.79% - -2.37%)	38.1% (32.8% − 43.4%)	60.6% (54.1% − 67.0%)
Institution-specific conversion factors	0.68% (0.46% − 0.90%)	68.3% (63.4% − 73.2%)***	86.9% (83.0% − 90.9%)***
Number of samples: 300			
Scoring system	1.29% (1.19% − 1.38%)	71.6% (67.2% − 76.1%)***	88.4% (85.5% − 91.3%)***
Conversion factor (0.64)	-2.59% (-2.72% - -2.46%)	40.3% (36.7% − 44.0%)	60.0% (55.5% − 64.5%)
Institution-specific conversion factors	0.73% (0.61% − 0.86%)	72.3% (67.9% − 76.6%)***	88.7% (84.9% − 92.5%)***
Number of samples: 400			
Scoring system	1.15% (1.04% − 1.27%)	73.1% (67.9% − 78.3%)***	90.3% (86.2% − 94.5%)***
Conversion factor (0.64)	-2.94% (-3.02% - -2.86%)	30.7% (25.2% − 36.2%)	56.2% (51.3% − 61.1%)
Institution-specific conversion factors	0.47% (0.40% − 0.54%)	74.8% (71.3% − 78.3%)***	89.7% (86.8% − 92.5%)***

*** *p *< 0.001, compared with the Conversion factor (0.64) group

When the small number of samples (100–400 samples per endoscopist) was used, the mean difference between estimated and actual ADR predicted using our developed scoring system varied from 1.15% to 1.40%, which indicates that the scoring system slightly overestimated the ADR of each endoscopist in conditions with small number of samples, the same to the performance of it in overall data. The proportion of estimations when the difference between estimated and actual ADR was less than 2% and 3% raised gradually from 56.7% to 71.6% and from 78.9% to 88.4%, respectively, as the number of samples increasing from 100 to 300. However, when the number of samples increased to 400, the proportion of acceptable estimations raised slightly and were similar with those in condition with 300 samples (Deviation ≤ 2%: 73.1%; Deviation ≤ 3%: 90.3%). In addition, in all small-sample settings, both our developed scoring system and the institution-specific conversion factors showed significantly higher proportions of acceptable estimations than the conversion factor (0.64), while the performance of our developed scoring system was comparable to that of the institution-specific conversion factors (our scoring system vs. institution-specific conversion factors vs. conversion factor (0.64); deviation ≤ 2%: 100 samples, 56.7% vs. 58.3% vs. 40.0%, *p* < 0.001; 200 samples, 66.4% vs. 68.3% vs. 38.1%, *p* < 0.001; 300 samples, 71.6% vs. 72.3% vs. 40.3%, *p* < 0.001; 400 samples, 73.1% vs. 74.8% vs. 30.7%, *p* < 0.001; deviation ≤ 3%: 100 samples, 78.9% vs. 78.9% vs. 60.8%, *p* < 0.001; 200 samples, 82.8% vs. 86.9% vs. 60.6%, *p* < 0.001; 300 samples, 88.4% vs. 88.7% vs. 60.0%, *p* < 0.001; 400 samples, 90.3% vs. 89.7% vs. 56.2%, *p* < 0.001).

These results indicate that our developed scoring system outperformed the recommended conversion factor (0.64) under more clinically relevant conditions with small sample sizes, while showing performance comparable to that of the locally calibrated benchmark, institution-specific conversion factor. In addition, our developed scoring system achieved its best practical performance when the number of samples was 300.

## Discussion

There is a paucity of published researches on investigating the relationship between ADR and PDR. A meta-analysis study including twenty-five studies and 31,623 patients from nine countries has demonstrated ADR was correlated with PDR and shown a APDRQ of 0.68 [12]. Some recent studies also showed high correlation between ADR and PDR, but different APDRQ varying from 0.64 to 0.73 [9, 13–17] . In subgroup analysis, A study investigating 2285 polyps removed from 1921 patients has shown that PDR and ADR correlated well in proximal colon, but diverged in distal colon [18]. However, another study has demonstrated high correlation between ADR and PDR in all segments of colon [15]. In order to verify the clinical value of APDRQ in estimating ADR from PDR, some studies further investigated the relationship between estimated and actual ADR predicted using APDRQ and suggested that the APDRQ can be used as a conversion factor to calculate ADR from PDR in the clinical practice [10, 13, 17]. Thus, the latest ESGE guideline recommended a ratio of 0.64 as conversion factor to predict the ADR from the PDR based on its good performance in a 3367 colonoscopies cohort performed by 20 endoscopists [9].

Though the strong correlation between PDR and ADR was proved in previous studies, seldom of them showed the deviation between estimated and actual ADR and none of them evaluated the level of agreement, a more important indicator, between estimated and actual ADR in each endoscopist. In addition, even though the good performance of APDRQ in estimating ADR from PDR was proved, the overall APDRQ in each center and APDRQ for each endoscopist in these studies varied largely, which may have a negative impact on generalization and practical significance of recommended APDRQ (0.64). Furthermore, none of previous studies focused on the performance of APDRQ in conditions with small number of samples, a more clinically relevant condition, and the establishment of lower limit of the number of colonoscopies on usage of APDRQ.

In our study, patient age, number of proximal or distal polyps, maximum size of proximal or distal polyps, and number of proximal or distal polyps with any size, except distal diminutive polyps, were significantly associated with pathologically confirmed adenoma detection in patients with polyp detection. Based on regression coefficients, we developed four optional scoring systems to predict adenoma detection in patients with polyp detection and finally chose the best one according to their performance on validation cohorts. Our developed scoring system slightly overestimated (about 1%) the ADR of each endoscopist, had higher level of agreement between estimated and actual ADR, and had a higher proportion of acceptable estimations when the deviation between estimated and actual ADR was less than 2%, compared with the recommended conversion factor (0.64). The outperformance of our developed scoring system was also verified in condition with small number of samples, which is more clinically relevant. Furthermore, based on the performance of our developed scoring system in conditions with small number of samples, 300 colonoscopies were considered as lower limit of the number of colonoscopies on usage of our developed scoring system. All of these makes our results more reliable and comprehensive.

In our study, we additionally introduced the institution-specific conversion factor as a locally calibrated benchmark to further assess the performance of our developed scoring system. The results showed that our developed scoring system performed comparably to the institution-specific conversion factor in both the overall analysis and the small-sample analysis, further supporting the robustness and validity of the model. However, the two approaches differ substantially in their practical requirements and intended use. Institution-specific conversion factors requires prior linkage between colonoscopy and pathology data within each institution to derive a local coefficient, and therefore depends on historical data availability and center-specific calibration. In contrast, our simplified scoring system was specifically designed for real-world settings in which direct integration with the pathology system is unavailable or difficult to implement. Importantly, our model does not require prior local calibration, yet it still performed robustly in both the development cohort and the external validation cohort across two independent centers. Therefore, although institution-specific APDRQ serves as a useful locally calibrated benchmark, our simplified scoring system may be more practical for broader clinical application.

The clinical applicability of our scoring system should also be interpreted in the context of the intended use scenario. In institutions with well-integrated pathology-endoscopy databases, directly calculated ADR remains the preferred standard, and the added value of estimation may be limited. Therefore, our model is not intended to replace directly measured ADR in such settings. Instead, the proposed scoring system may be most useful in units where linkage between endoscopic and pathological data is unavailable, incomplete, or difficult to implement routinely. In these circumstances, the model may serve as a simplified and pragmatic tool for endoscopist-level quality monitoring. It may also have value when more timely feedback is required, such as during routine quality audits before full pathology linkage is completed. In addition, because the model relies only on routinely available endoscopic findings and basic patient information, it may be more feasible in multicenter, community-based, or resource-limited settings where automated ADR calculation cannot be easily achieved.

Although our developed scoring system may be clinically useful, the current study has several limitations. Although our developed scoring system may be clinically useful, the current study has several limitations. First, some important factors that may affect adenoma detection in patients with polyp detection, such as colonoscopy indication and the use of magnifying or image-enhanced endoscopy, were not completely recorded in our electronic database, which may have reduced the accuracy of the model. However, the scoring system was intentionally designed to rely only on patient age and simple endoscopic descriptions of detected polyps, including location and size, which may improve its feasibility in routine clinical practice. Second, other factors known to influence polyp and adenoma detection, such as withdrawal time and the endoscopist’s experience, were not included because they could not be accurately or consistently obtained for all included examinations in this retrospective database-based study. Their omission may have introduced residual confounding. In addition, ADR is context-dependent and may be influenced by patient case-mix and colonoscopy indication, in addition to procedural performance. Although we reduced heterogeneity through the exclusion criteria, residual variation in case-mix may still have affected both ADR and model performance. Third, histopathological misclassification in diminutive lesions may have occurred because sampling limitations, tissue sectioning, and interobserver variability can affect pathological classification, particularly in distal diminutive polyps. Therefore, the lower adenoma rate observed in these lesions may reflect not only true biological differences, but also diagnostic limitations. Fourth, non-adenomatous lesions were analyzed as a single category. Although this group includes lesions with distinct biological behavior, the primary aim of this study was to estimate the conversion from polyp detection to adenoma detection rather than to distinguish pathological subtypes of non-adenomatous lesions, and this simplification may limit the biological interpretation of the observed conversion patterns. Fifth, the model was developed and validated exclusively in two Chinese centers. Although external validation in an independent second-center cohort supports a certain degree of robustness, differences in demographics, ethnicity, endoscopic equipment, procedural patterns, and operator expertise may limit its generalizability to other populations and practice settings. Finally, this was a retrospective and observational study, and selection bias and overfitting may still exist. Further prospective multicenter studies are needed to validate the performance and generalizability of this newly developed scoring system.

## Conclusion

In conclusion, our developed 5-item scoring system based on significant predictor for pathologically confirmed adenoma detection in patients with polyp detection (age ≥ 70 years, positive; proximal polyps with any size, positive; distal polyps ≥ 10 mm, positive; age 60 ~ 69 years, 1 point; per distal small polyps, 1 point; pathologically confirmed adenoma detection was predicted when a patient met any positive criterion or, in the absence of these positive items, had a total score > 1 point) outperformed the recommended conversion factor (0.64) and predicted ADR from PDR of each endoscopist accurately in both overall data and more clinically relevant condition with small number of samples. Additionally, 300 colonoscopies per endoscopist was the best number of samples when this scoring system was used in clinical practice.

## Data Availability

The data that support the findings of this study are available from the corresponding author, upon reasonable request.

## Abbreviations

ADR: Adenoma detection rate
CRC: Colorectal cancer
ESGE: European Society Gastrointestinal Endoscopy
PDR: Polyp detection rate
APDRQ: Adenoma to polyp detection rate quotient
ROC: Receiver operating characteristics
AUROC: Area under the receiver operating characteristics curve
LOA: Limit of agreement
